# Role of Glomerular Proteoglycans in IgA Nephropathy

**DOI:** 10.1371/journal.pone.0018575

**Published:** 2011-04-06

**Authors:** Kerstin Ebefors, Anna Granqvist, Madeleine Ingelsten, Johan Mölne, Börje Haraldsson, Jenny Nyström

**Affiliations:** 1 Department of Molecular and Clinical Medicine – Nephrology, Institute of Medicine, The Sahlgrenska Academy, University of Gothenburg, Gothenburg, Sweden; 2 Institute of Biomedicine, Department of Infectious Medicine, The Sahlgrenska Academy, University of Gothenburg, Gothenburg, Sweden; 3 Department of Pathology, Institute of Biomedicine, The Sahlgrenska Academy, University of Gothenburg, Gothenburg, Sweden; 4 Department of Physiology, Institute of Neuroscience and Physiology, The Sahlgrenska Academy, University of Gothenburg, Gothenburg, Sweden; University of Houston, United States of America

## Abstract

Mesangial matrix expansion is a prominent feature of the most common form of glomerulonephritis, IgA nephropathy (IgAN). To find molecular markers and improve the understanding of the disease, the gene and protein expression of proteoglycans were investigated in biopsies from IgAN patients and correlated to clinical and morphological data. We collected and microdissected renal biopsies from IgAN patients (n = 19) and from healthy kidney donors (n = 14). Patients were followed for an average time of 4 years and blood pressure was according to target guidelines. Distinct patterns of gene expression were seen in glomerular and tubulo-interstitial cells. Three of the proteoglycans investigated were found to be of special interest and upregulated in glomeruli: perlecan, decorin and biglycan. Perlecan gene expression negatively correlated to albumin excretion and progress of the disease. Abundant decorin protein expression was found in sclerotic glomeruli, but not in unaffected glomeruli from IgAN patients or in controls. Transforming growth factor beta (TGF-β), known to interact with perlecan, decorin and biglycan, were upregulated both on gene and protein level in the glomeruli. This study provides further insight into the molecular mechanisms involved in mesangial matrix expansion in IgAN. We conclude that perlecan is a possible prognostic marker for patients with IgAN. In addition, the up-regulation of biglycan and decorin, as well as TGF-β itself, indicate that regulation of TGF-β, and other profibrotic markers plays a role in IgAN pathology.

## Introduction

IgA nephropathy (IgAN) is a poorly understood disease with a largely unknown molecular background. It is the most common type of glomerular nephritis and, although it is considered benign, the majority of patients will eventually develop chronic kidney disease stage V. Hence, it is of vital importance to understand the pathogenesis in order to predict the risk of progression and improve treatment strategies. Morphologically, IgAN is characterized by the presence of immunoglobulin A (IgA) deposits in the mesangial region, proliferation of mesangial cells and expanded mesangial matrix [1]. The mesangial matrix is synthesized by mesangial cells and consists of a mix of glycoproteins and various negatively charged proteoglycans (PGs) [2]. Proteoglycans are complex molecules with properties determined by their glycosaminoglycan chains as well as their core protein. Their functions range from structural roles in the extracellular matrix to involvement in cell signaling, both by acting as binding sites, controlling growth factor gradients, and as signaling molecules [3], [4]. We have previously investigated the role and function of proteoglycans in various diseases and disease models and found them to be of importance both for the development of nephrotic syndrome and normal function of the glomerular filtration barrier [5], [6], [7], [8], [9]. Proteoglycans also contribute significantly to the charge-selective properties of the barrier [10], [11], although debated [12]. PGs occur not only in the mesangial matrix but also in the glomerular endothelial glycocalyx [7], [8], [13], the basement membrane [14], and the podocytes [9]. In IgAN, PGs are thought to be of pathophysiological relevance, both as biomarkers and actually affecting clinical outcome of the disease [15], [16], [17], [18]. In the present study, we investigated the gene expression of PGs and PG modulators, separately in the glomerular and tubular parts of kidney biopsies. The expression of transforming growth factor beta (TGF-β), nephrin and VEGF was investigated as well. TGF-β and VEGF have both been implicated to play a role in IgAN and they are also known to interact with PGs [19], [20], [21]. Nephrin is a protein crucial for podocyte function and damage and therefore of interest in IgAN [22]. We then linked the gene expression results to clinical and morphological data in order to learn more about the underlying molecular mechanisms of IgAN.

## Results

### Characteristics of patients

Clinical data at the time of biopsy are presented in table 1. All patients' progress and mean arterial blood pressure were followed for an average time period of 4 years. All patients with IgAN had a well-maintained blood pressure during the follow-up period, and there was no correlation between the progress of the disease and the mean arterial blood pressure.

**Table 1:** Clinical characteristics of patients with IgAN at time of biopsy.

patient ID	Sex	Age	GFR	Serum creatinine (µmol/L)	Serum albumin (g/L)	Albumin excretion (mg/24H)	MAP (mm Hg)	BP medication	RAAS inhibitors
1	M	33	61	149	41	-	118	Yes	Yes
2	M	37	97	105	41	18	95	No	No
3	F	41	91	89	29	178	107	No	No
4	M	31	99	84	47	654	107	No	No
5	F	36	111*	54	37	334	95	No	No
6	F	30	80	73	37	1100†	86	Yes	Yes
7	M	47	63	117	40	1081	93	Yes	Yes
8	F	40	76	114	37	1100	88	Yes	Yes
9	M	24	100*	82	30	335	82	No	No
10	M	60	23	222	31	1100†	93	No	Yes
11	F	43	85*	66	34	799	77	No	No
12	M	57	51	104	39	690	110	Yes	Yes
13	M	66	24	160	39	83	105	Yes	Yes
14	M	55	61*	109	46	34	107	Yes	Yes
15	M	40	60*	117	39	392	102	Yes	No
16	M	49	97	124	34	1029	95	No	No
17	M	35	65	109	36	1119	104	No	No
18	M	33	45	124	44	71	103	Yes	Yes
19	M	69	47	153	33	1991	95	Yes	Yes

Abbreviations; GFR, glomerular filtration rate; MAP, mean arterial pressure; BP, blood pressure; RAAS, Renin-angiotensin-aldosterone system.

*estimated GFR calculated using MDRD formula (ml/min/1.73 m^2^), the other GFRs presented in the table are determined using ^51^Cr-EDTA clearance.

† calculated from tU-protein.

### Gene expression in the glomerular and tubulo-interstitial compartments

**Figure 1:**
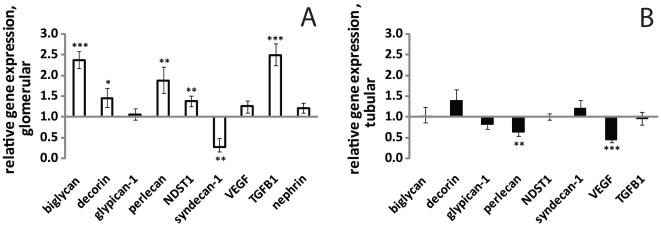
Relative gene expression of proteoglycan core proteins and enzymes in glomeruli (A) and tubulo-interstitial compartment (B) of biopsies from IgAN patients compared to controls. Controls values are defined as 1, and higher values indicate up-regulation while lower values indicate down-regulation. Error bars represent SEM. *p<0.05, **p<0.01, ***p<0.001.

The expression levels of several of the PGs and PG related genes investigated were altered (figure 1) in patients with IgAN compared to healthy controls. More of the investigated genes were affected, and to a higher degree, in the glomerular than the tubulo-interstitial compartment of the biopsy.

#### Glomerular gene expression in IgAN

Perlecan expression in glomeruli was increased by 87.0% (+33.8, −28.6, p<0.01, n = 18). Glomerular expression of the two small leucine rich PGs, biglycan and decorin, was increased by 136.6% (+21.9, −20.0, p<0.001, n = 18), and 45.1% (+23.9, −20.5, p<0.05, n = 18), respectively. The enzyme N-deacetylase/N-sulfotransferase 1 (NDST1), crucial for heparan sulfate production, increased by 37.6% (+13.4, −12.2, p<0.01, n = 18). In addition, TGF- β was elevated by 149.6% (+27.2, −24.6 p<0.001, n = 12) compared to control. Glomerular gene expression of the proteoglycan syndecan-1 was decreased by −72.1% (+20.6, −11.9 p<0.01 n = 18). Glomerular nephrin expression did not change significantly.

#### Tubular gene expression in IgAN

Tubular perlecan expression was decreased by −38.2% (+9.7, −8.4, p<0.01, n = 15), while no significant changes were found in NDST1, TGF-β, biglycan, decorin and syndecan-1. Expression of vascular endothelial growth factor (VEGF) was significantly reduced, −56% (+6.2, −5.4, p<0.001 n = 18), in the tubulo-interstitial part of the biopsy.

### Correlation of glomerular and tubular mRNA expression

**Figure 2:**
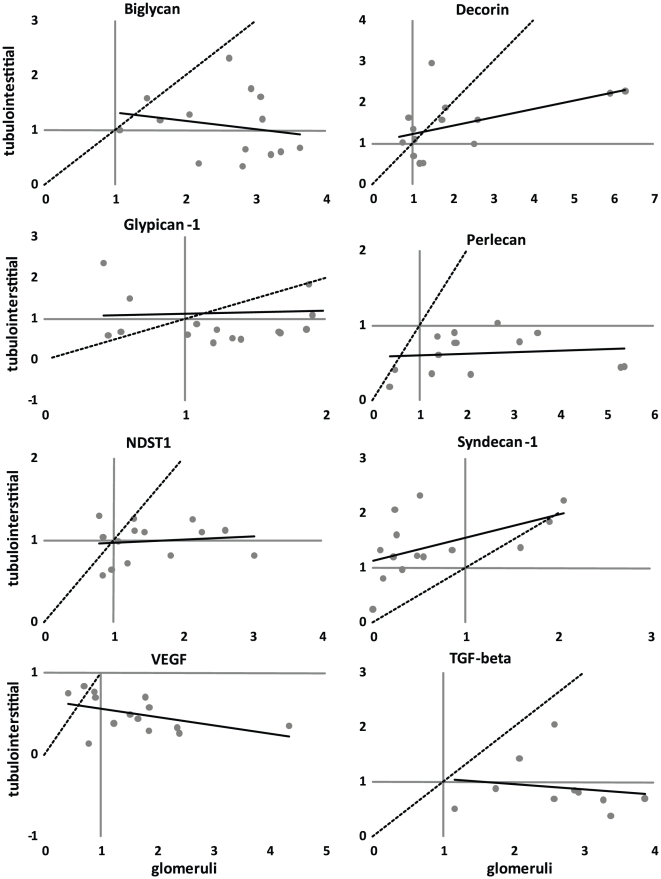
Relative gene expression in the tubulo-interstitium and the glomeruli. Microdissection makes it possible to plot the glomerular and tubulo-interstitial values against each other. The expected outcome of identical glomerular and tubulo-interstitial expression is shown as a dotted line. No correlations between tubular and glomerular gene expression were found, indicating that glomerular and tubulo-interstitial gene expression is independent of each other.

The investigated genes showed completely different expression patterns in glomerular and tubulo-interstitial cells. This is illustrated in figure 2.

### Correlation between gene expression and clinical parameters

**Figure 3:**
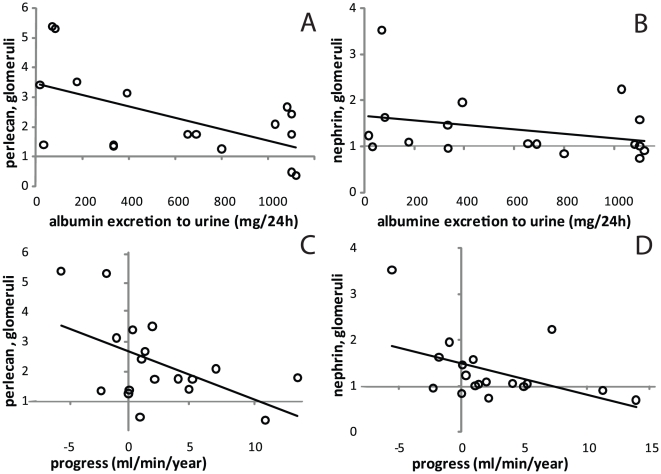
The relative gene expression of perlecan in the glomeruli from patients with IgAN at time of the biopsy correlates inversely to the patient's albumin excretion in urine, n = 17, r = −0.58, p<0.05 (A) and disease progression, n = 18, r = −0.52, p<0.05 (C). A high expression of perlecan at the time of the biopsy correlates to a lower albumin excretion and slower progression of the disease, implicating that the relative gene expression of perlecan can be used as a prognostic molecular marker in IgAN. The relative gene expression of nephrin did not correlate to urinary albumin excretion, n = 17 (B), but correlated inversely to disease progression, n = 18, r = −0.47, P<0.05 (D). This suggests that even the smallest changes in gene expression of nephrin may be important for disease progression in IgAN.

Gene expression levels in IgAN patients were correlated to the two clinical parameters; proteinuria and progress rate. Proteinuria was inversely related to perlecan expression in glomeruli (r = −0.58, p<0.05 n = 17) (figure 3). It also correlated to tubular mRNA expression for biglycan (r = 0.59, p<0.05 n = 14) and decorin (r = 0.64, p<0.05 n = 14). The progress rate of the disease, calculated from patient creatinine clearance, inversely correlated to the glomerular expression of perlecan mRNA (r = −0.52, p<0.05 n = 18) and nephrin mRNA (r = −0.47, P<0.05 n = 18), figure 3.

### Expression of decorin, TGF-β and perlecan protein

**Figure 4:**
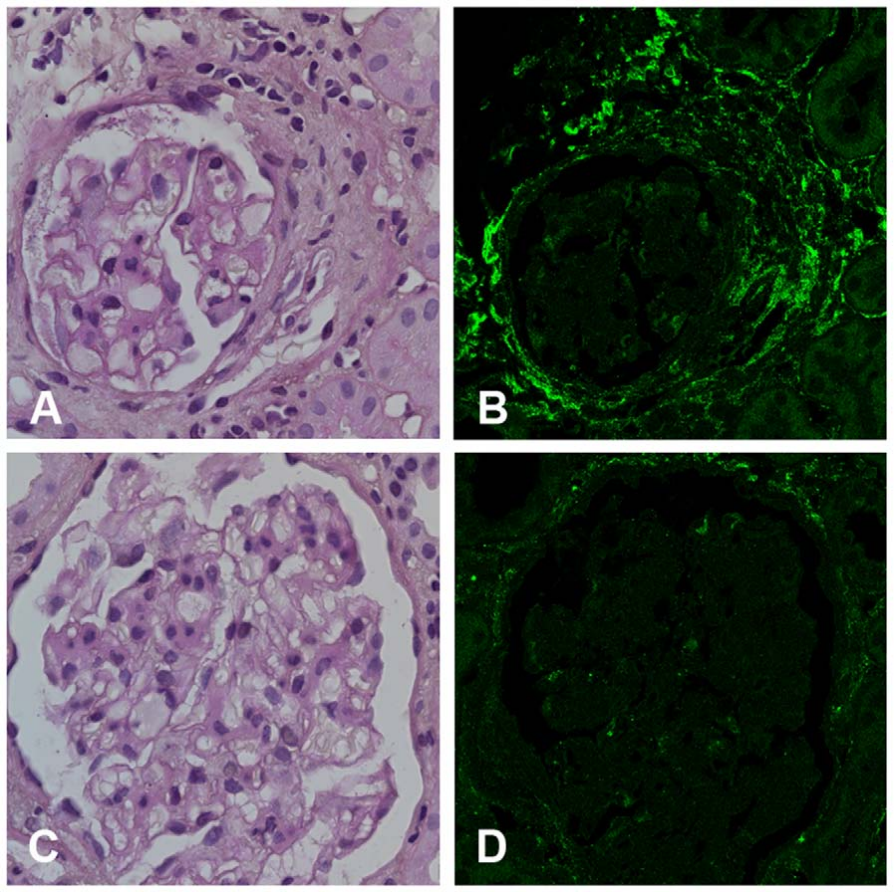
PAS staining of sclerotic glomeruli from a representative IgAN patient (A), and the consecutive section stained for decorin (B) shows overlap of the sclerotic area and decorin staining. PAS staining of glomeruli without sclerosis (C) and consecutive section showing no staining for decorin (D) in the same patient. Decorin is abundantly expressed in sclerotic glomeruli both in controls and patients with IgAN, but not in non-sclerotic glomeruli. Magnification ×63.

**Figure 5:**
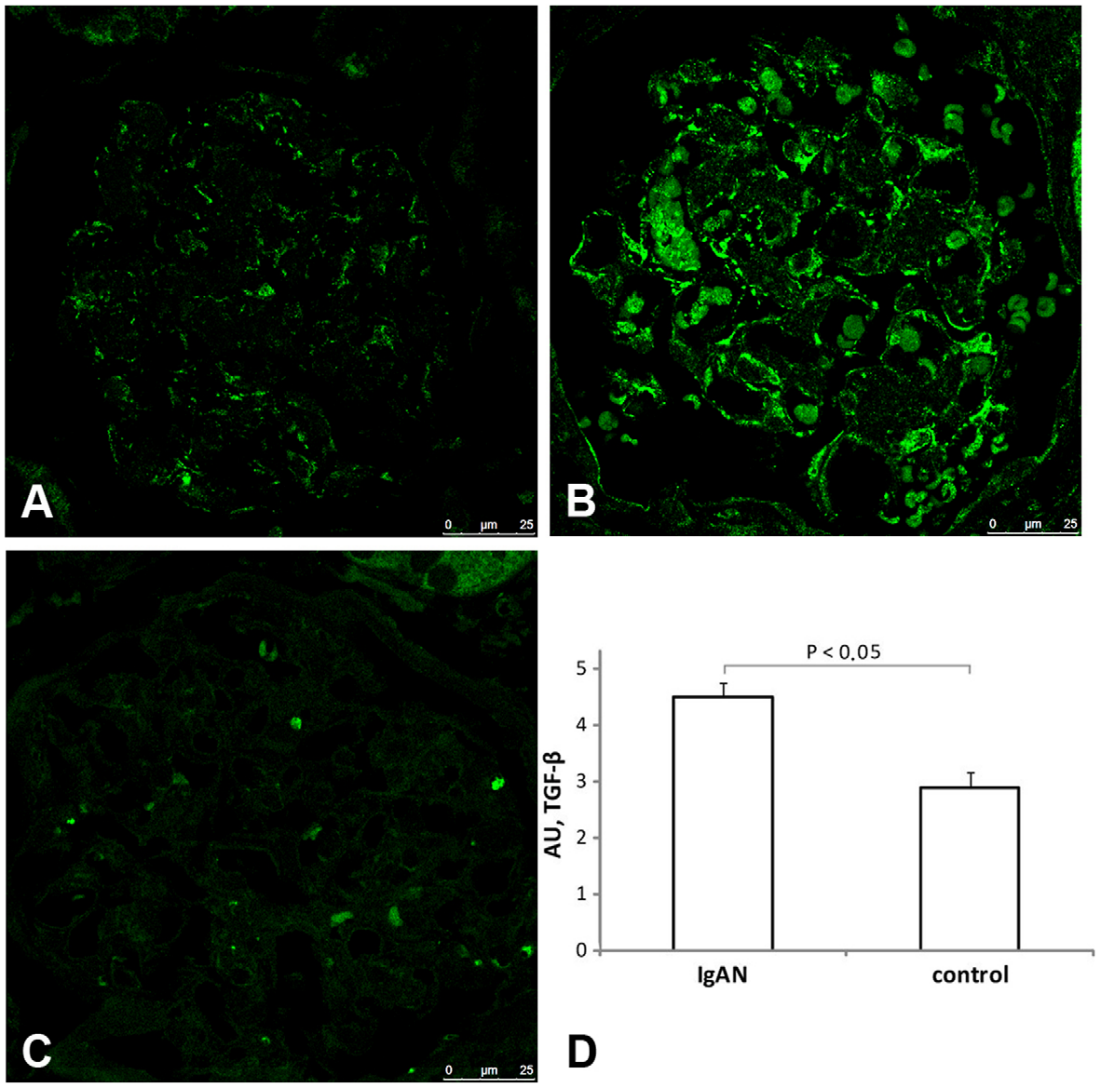
Protein expression of TGF-β in sections from control (A), patient with IgAN (B) and negative control (C). IgAN sections stained significantly stronger for TGF-β, and the average arbitrary unit score for IgAN (n = 83 glomeruli, 4.51±0.25) was higher than control (n = 19 glomeruli, 2.89±0.28) P<0.05 (D). In (B) there is some auto-fluorescence of red blood cells, this phenomenon can also be seen in the negative control, and were not included in the analysis of staining intensity of TGF-β. Magnification ×63.

**Figure 6:**
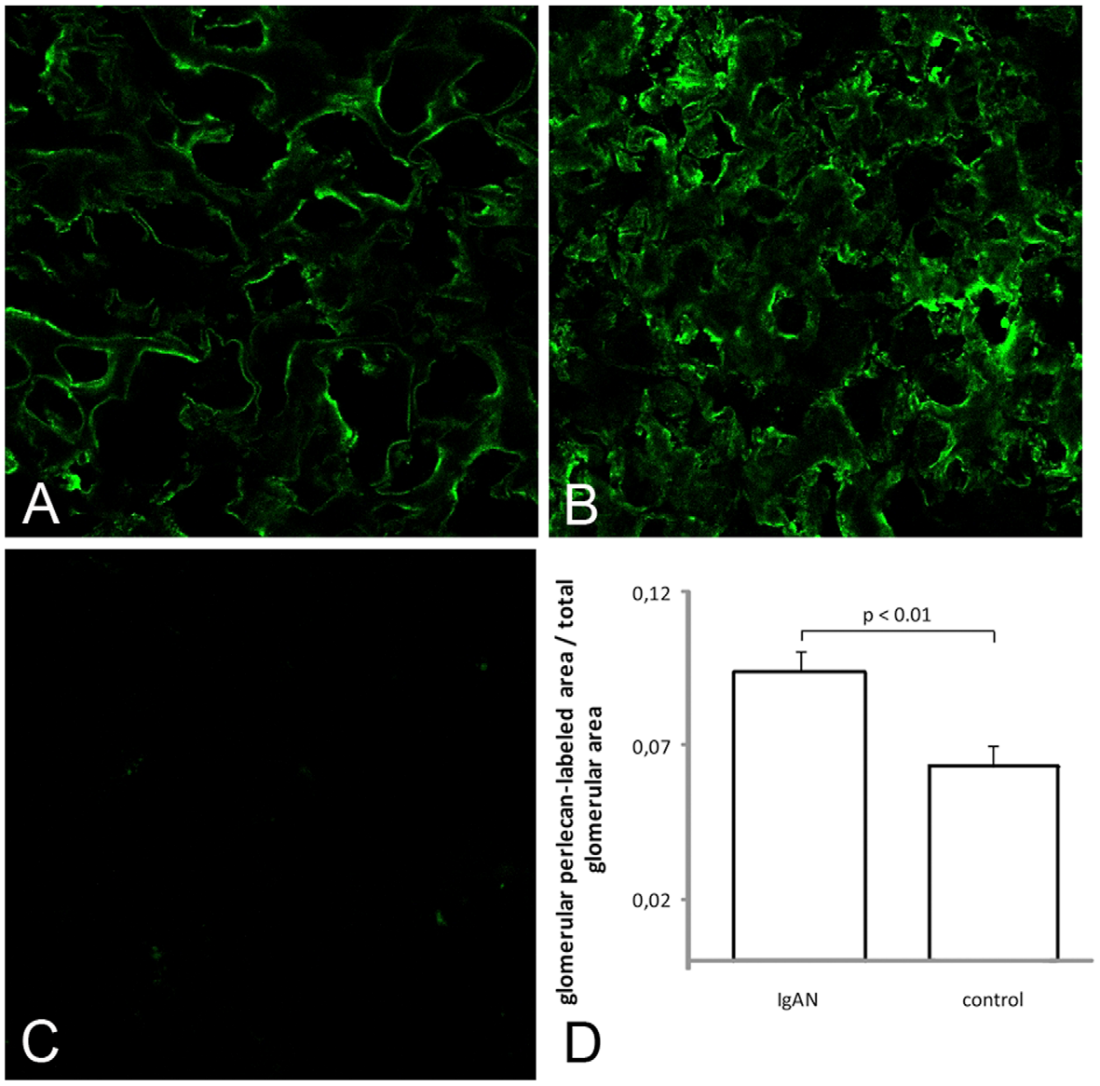
Protein expression of perlecan in sections from control (A), a patient with IgAN (B), and a negative control (C). Protein expression of Perlecan in frozen biopsy sections from another set of patients with IgAN stained significantly stronger for perlecan (n = 45, 6.3±0.7%) than controls (n = 30, 9.4±0.7%) P<0.01. Magnification ×63.

To confirm propagation of mRNA changes to the protein level, immunofluorescence studies were performed on paraffin sections from IgAN and control biopsies. There was no immunofluorescence staining for decorin in histologically normal glomeruli from either control or IgAN patients, but sclerotic glomeruli stained for decorin in both groups, see figure 4 for expression in an IgAN patient. There was also staining for decorin in the renal interstitium in both IgAN and controls, but no detectable difference between the groups, data not shown. IgAN glomeruli stained more intensely for TGF-β compared to controls, as seen in figure 5. The average arbitrary unit score for IgAN (n = 83, 4.51±0.25) was significantly higher than control (n = 19, 2.89±0.28), P<0.05. Immunofluorescence studies of perlecan were performed on frozen biopsy sections from another set of patients and controls, see figure 6. The staining was more intense in glomeruli from patients with IgAN (n = 45, 9.4±0.7%) than controls (n = 30, 6.3±0.7%), P<0.01.

### Oxford classification

Patients with IgAN were classified using the Oxford classification system according to the international IgA Nephropathy Network and the Renal Pathology Society [23], [24]. By transforming the pathologists primary data into a scoring system, shown in table 2, patients were grouped according to a mesangial hypercellularity score of ≤0.5 (M0) or >0.5 (M1), without (E0) or with (E1) endocapillary hypercellularity, and without (S0) or with (S1) segmental glomerulosclerosis and tubular atrophy/interstitial fibrosis (TA/IF), 0–25% (T0), 26–50% (T1) and >50% (T2).

**Table 2:** Summary of key pathological features according to the Oxford classification system.

Patient ID	Mesangial score	Segmental GS	Endocapillary hypercellularity	TA/IF
1	1	0	0	0
2	0	0	0	0
3	0	0	0	0
4	0	1	0	0
5	1	1	0	0
6	1	1	1	0
7	1	1	1	0
8	1	1	0	1
9	1	1	1	0
10	1	0	1	2
11	0	0	0	0
12	0	1	1	1
13	1	1	0	1
14	0	0	0	1
15	1	1	1	0
16	0	1	1	1
17	1	1	1	1
18	0	1	0	1
19	1	1	1	2

Mesangial score ≤0.5 (M0) or >0.5 (M1).

Segmental glomerulosclerosis, absent (S0) or present (S1).

Endocapillary hypercellularity, absent (E0) or present (E1).

TA/IF ≤25% (T0), 26–50% (T1), or >50% (T2).

### Correlation between gene expression and Oxford classification data

The percentage of glomeruli with global glomerulosclerosis in each biopsy specimen correlated to the expression of the proteoglycan glypican-1 in the glomerular fraction (r = 0.57, n = 18, p<0.05). When classifying the patients according to absence or presence of endocapillary hypercellularity, glomerular glypican-1 expression was significantly different between the groups (E0: 45.4%±17.8 n = 10 vs E1: −9.8%±15.8 n = 8, p<0.05). Similarly, glomerular gene expression of VEGF was higher in the group with segmental glomerulosclerosis (S1: 74.9%±29.4, n = 12), than in the group without (S0: −3.7%±15.4, n = 6, p<0.05).

When comparing the gene expression data from the tubulo-interstitial compartment to the TA/IF score, a correlation was found for four of the proteoglycans. Thus, TA/IF correlated to the expression of biglycan (n = 15, r = 0.61, p<0.05), decorin (n = 15, r = 0.61, p<0.05), perlecan (n = 15, r = 0.52, p<0.05) and glypican-1 (n = 15, r = 0.57, p<0.05) as well as the enzyme NDST1 (n = 15, r = 0.65, p<0.01) and TGF-β (n = 15, r = 0.76, p<0.001). Decorin (n = 15, r = 0.64, p<0.05) and glypican-1 (n = 15, r = 0.79, p<0.001) gene expression in the tubulo-interstitium also correlated to presence of global glomerulosclerosis. Patients with extracapillary proliferation had a higher NDST1 gene expression (n = 15, r = 0.52, p<0.05). Finally, when dividing TA/IF into two groups and studying VEGF expression, the groups differed significantly (T0 −37.7%±7.9, n = 8, T1+T2 −66.1%±2.3, n = 7, p<0.05).

### Correlations between clinical and Oxford classification data

**Figure 7:**
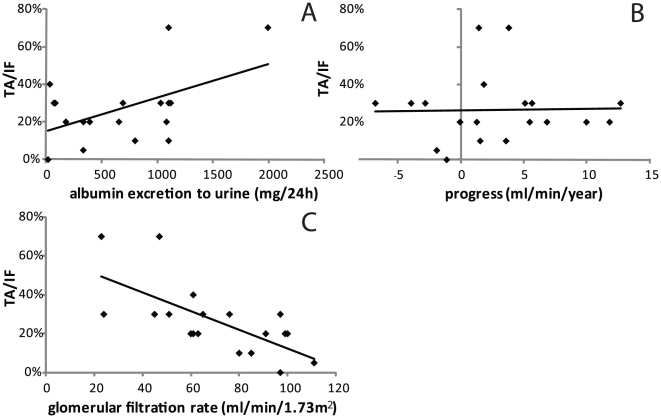
Correlation of tubular atrophy/interstitial fibrosis (TA/IF) score and albumin excretion in urine, r = 0.51, p<0.05 (A), progress, non-significant (B) and glomerular filtration rate, r = −0.68, p<0.01 (C).

Clinical data from the IgAN patients were compared to the Oxford classification system. Glomerular filtration rate (GFR) (r = −0.68, p<0.01 n = 19), urinary albumin excretion (r = 0.51, p<0.05 n = 18), but not the progression of the disease, correlated to TA/IF, see figure 7. GFR was negatively correlated to the percentage of global glomerulosclerosis (r = −0.47, P<0.05 n = 19) and albumin excretion correlated with presence of segmental glomerulosclerosis (r = 0.54, n = 18, p<0.05) and extracapillary proliferation (r = 0.59, n = 18, p<0.01). There was a significant difference in GFR between patients with low (0–25%, T0) or high (>25%, T1 and T2) levels of TA/IF (84.7±5.8, n = 10, compared to 54.3±7.9, n = 9 (7 of T1 and 2 of T2), p<0.05). There was also significant differences between the groups, when studying albumin excretion in urine, and dividing patients with mesangial hypercellularity into those with scores ≤0.5 (M0) or >0.5 (M1) (434±142, n = 8, vs. 864±181, n = 10, p<0.05). Albumin excretion was also different in the two endocapillary hypercellularity groups, without (E0) or with (E1), 363±131, n = 9, vs. 982±164, n = 9, p<0.01.

## Discussion

This is the first report on glomerular and tubular gene and protein expression of proteoglycans (PGs) and PG related genes in micro-dissected biopsies from patients with IgA nephropathy (IgAN). The gene expression data were correlated to clinical data and morphological scores. Three important conclusions can be drawn from our study: Firstly, there is an increased glomerular mRNA expression of several PGs in biopsies from IgAN patients. Also, expression of NDST1 was increased. NDST1 is an enzyme essential for adding sulphate (and thereby negative charge) to the glycosaminoglycan chains of perlecan [25] and other PGs. TGF-β, a pro-fibrotic growth factor known to affect PGs was up-regulated as well. Secondly, tubular cells demonstrate a different pattern, with down-regulation of perlecan as well as VEGF, while all other investigated genes were unaffected. Thus, our results clearly show that the composition of glomerular matrix PGs is altered in patients with IgAN. Thirdly, these results correlate with clinical data, where increased perlecan expression correlates with a better outcome for the patient (less albumin excretion in urine, slower progression of disease and a lower TA/IF score).

Perlecan is a highly negatively charged PG found at all levels of the filtration barrier [8], [9], and is one of the most common PGs in the basement membrane [26]. In this study, patients with IgAN had an increased expression of perlecan in the glomerular portion, and a reduced gene expression in the tubulo-interstitial portion of the biopsy. The increased expression of perlecan in glomeruli was confirmed on the protein level. The group of van den Born et al. found an increased expression of perlecan in the mesangial area in IgAN compared to control, but not in the basement membrane [27]. We found that patients with an increased perlecan expression in glomeruli had a lower urinary albumin excretion and a slower progress of the disease. These data from our study indicate that perlecan is a possible prognostic marker for patients with IgAN, and that patients with high perlecan expression have slower deterioration of renal function and therefore better prognosis. This, however, might not be due to the charge-selective properties of perlecan. Both the perlecan core protein and the GAG chains are able to interact with diverse extracellular matrix molecules, such as basement membrane components, growth factors and receptors [28] [29]. The increase in perlecan could be an effort to regenerate the function and structure of the matrix in the glomeruli. Earlier studies of the amount of anionic sites in IgAN have shown a decrease in anionic sites [30], [31], or an unaltered staining for the heparan sulfate chains [27]. The negative charge of all PGs comes from the sugar-based side chains to the core protein. We have only investigated the core protein and not the charge and number of side chains. Such changes in the carbohydrate moiety could of course lead to substantially altered properties of PGs.

Another important protein for regulating matrix composition is TGF-β. We found a 2.5 fold increase of glomerular TGF-β gene expression in patients with IgAN, and this was confirmed on protein level. Renal TGF-β production is considered to be increased in IgAN [21] and diabetic nephropathy [32]. In the gene expression study of Waga et al, where biopsies from 12 patients were analyzed without microdissection, TGF-ß was unchanged to slightly down-regulated for mild to severe IgAN [33]. This is in agreement with our findings for the expression in the tubulo-interstitial compartment, which indeed is known to represent 90–95% of renal cortex mRNA. Thus, without microdissection the up-regulation of glomerular TGF-β seen in the present study would have been undetected. Mesangial cells in the glomerulus are affected by TGF-β, and podocytes treated with TGF-β increase their production of biglycan as well as fibronectin and type IV collagen [34]. In an animal model of acute mesangial proliferative glomerulonephritis, the injured glomeruli expressed more TGF-β and synthesized more fibronectin and proteoglycans than normal glomeruli [35]. The TGF-β induced deposition of extracellular matrix can lead to scarring and fibrosis. Thus, the increased TGF-β we found in patients with IgAN may be one pathological factor that leads to altered matrix production and composition.

The small leucine rich proteoglycans (SLRPs), decorin and biglycan, which we found to be upregulated in glomeruli in IgAN patients, have been suggested to play a major role in modulating the activity of growth factors. Decorin neutralizes the activity of TGF-β [36] and both biglycan and decorin can bind TGF-β [37]. Decorin is also thought to protect against the progression of diabetic kidney disease [38]. In a model of anti-Thy-1-initiated glomerulonephritis, injections with decorin suppressed the TGF-β activity [39]. Decorin, and its increased mRNA expression in IgAN, can therefore be protective for the glomeruli. In normal adult human kidneys only trace amounts or no protein expression of decorin has been found in glomeruli [40], [41], and this is in accordance with our findings. However, in glomeruli showing sclerosis we found abundant staining for decorin, see figure 4, this was seen both in glomeruli from patients with IgAN and normal controls. Decorin has been demonstrated at sites of glomerular fibrosis [41] and at elevated levels in the skin from patients with nephrogenic systemic fibrosis [42]. The other SLRP, biglycan, can also bind TGF-β and in addition toll-like receptor 4. The latter protein is important for the innate immune response and is increased in leukocytes from patients with IgAN [43]. Biglycan is one of the key predictive proteins for kidney disease progression found by Ju et al. [18] The upregulation of decorin and biglycan seen in this study indicates that SLRPs are also important in IgAN.

When correlating gene expression data with the clinical parameters, not only perlecan but also nephrin strongly correlated with disease progression. Nephrin is a podocyte-specific protein situated in the slit diaphragm between the podocyte foot processes and is vital for a maintained glomerular permselectivity [44]. The correlation of the gene expression of nephrin with the progress of the disease might be due to loss of podocytes as presented by Xu et al in patients with IgAN [45] or the podocyte flattening sometime seen in IgAN [46] [47], confirming that even small changes in nephrin gene expression may be of importance.

In order to improve diagnostic precision, and to enable forecasting of individual patient outcomes, the Oxford classification of IgAN was introduced in 2009. We used this classification system [23], [24] to compare gene expression and clinical data from our patients. In our patient group, the most powerful parameter in terms of predicting clinical outcome was the tubular atrophy/interstitial fibrosis (TA/IF) score. TA/IF correlates well with tubular expression data for perlecan, biglycan, decorin, glypican-1, NDST1 and TGF-β. Patients with higher TA/IF scores had reduced GFR. This reveals that not only glomerular anomalies, but also morphological alterations in the tubular parts of the nephron, are important for the development of proteinuria. Although IgAN is considered a glomerular disease, the damage occurring in the glomerulus with matrix expansion and sclerosis ultimately leads to tubular damage.

In conclusion, glomerular gene expression of proteoglycans was markedly changed in patients with IgAN. These changes correlated with clinical and morphological data showing that proteoglycans are important in the development and progress of IgAN, probably by alterations in the composition and production of the mesangial matrix. Further studies of molecular markers, such as perlecan and biglycan, are required to shed more light on the underlying mechanisms causing IgAN.

## Materials and Methods

### Ethic statement

After written informed consent and approval by the local ethical board of West Sweden (653-05, S 552-02, R110-98), one extra kidney biopsy was obtained from patients with renal disease, healthy living kidney donors and healthy parts from nephrectomized kidneys.

### Patients and controls

Biopsies were placed in RNAlater, refrigerated for 24 hours and then frozen in −20°C. The material was grouped according to diagnosis based on routine pathological analysis. Biopsies from patients with IgA nephropathy were then singled out (n = 19, age 43.5±12.8, female to male ratio 5∶14). Clinical characteristics are found in table 1. Biopsies from kidney transplants (n = 11) and normal tissue margins of nephrectomized kidneys (due to tumors, n = 3) were used as controls (n = 14, age 52.5±10.8, female to male ratio 1∶13). All patients with IgAN and all kidney donors had a well maintained blood pressure.

### Clinical data

The clinical data collected at the time of the renal biopsy included age, sex, GFR, serum albumin, serum creatinine, albumin excretion, mean arterial pressure and any antihypertensive medications used (Table 1). Serum creatinine values from an average period of 4.0 years (range; 1.9 to 7.1 years, 4–48 values with a mean of 15.8±12) starting 3 months before the biopsy was used to calculate creatinine clearance, and then the progress of the disease over time, using the Cockcroft-Gault equation (Rowland M. Clinical Pharmacokinetics, concepts and applications. 3rd edn., 1995). Blood pressure values collected during 3.5 years (range 0.2 to 6.7 years) were used for calculation of the mean arterial pressure. The MDRD formula [48] was used for calculating the estimated GFR in patients that had no measurement of GFR around the time of the biopsy [49].

### Isolation of RNA

Microdissection of biopsies stored in RNAlater was performed manually under a stereomicroscope to separate glomeruli from the tubulo-interstitial part of the biopsy. This was performed at a few time points and the material had been stored at −80°C for different time periods depending on the collection date of the biopsy. RNA was extracted from the glomeruli with RNeasy Micro kit and from the tubulo-interstitial part of the biopsy with RNeasy Mini kit (Qiagen, Hilden, Germany). The Agilent 2100 bioanalyzer, RNA Pico and Nano LabChip (Agilent technologies, Waldbronn, Germany), was used to determine the concentration and quality of the RNA. Since only high quality RNA was used in further steps, the number of glomerular samples in the gene expression studies were n = 18 (one sample showed degradation and was excluded), and for tubuli n = 15 (4 samples were degraded and excluded).

### Real time PCR

Reverse transcription of the RNA was performed in avian myeloblastosis virus reverse transcriptase (AMV RT) with AMV RT, dNTP, random hexamers and RNase inhibitor (Roche, Basel, Switzerland) in a final volume of 20 µl. The reaction was carried out at 25°C for 5 min, 42°C for 50 min and 70°C for 5 min on GenAmp PCR system 2700 (Applied biosystems, Foster city, CA). The mRNA level was quantified by real time PCR on the ABI Prism 7900 Sequence Detection system (Applied Biosystems) with low density arrays as described previously allowing analyses of several genes at the same time with as little as 10 ng of cDNA without amplification [9]. Three different setups of low density arrays were used, the first one with 23 different genes analyzed in duplicate in one run. The second array setup runs 16 different genes in triplicate by the same principle and the third array setup had 48 genes in singles. GAPDH was selected as endogenous control as it proved most stable, which is consistent with the literature [50]. The software allows combination of results run on different setups of arrays if the same genes are used. The comparative ΔΔC_T_ method in the software (Applied Biosystems) was used to calculate the difference in gene expression between controls and patients with IgAN. The entire control group was used as calibrator sample.

### Tissue processing and Oxford classification of IgA nephropathy

For light microscopy, serial paraffin sections, 3 µm in thickness, were produced at three levels. All biopsy specimens were stained with Masson trichrome, eosin, elastin, Jones silver and periodic acid Schiff (2 slides), a total of 18 sections for each biopsy. Separate sections were digested in protease, immuno-stained using a panel of antibodies to IgG, IgA, IgM, lambda, kappa, C1q, C3c, C5b-9 and fibrinogen/fibrin (all from Dako A/S, Copenhagen, Denmark) and processed in a computer-controlled immunostainer, TechMate 500 (Dako) using a detection kit, Dako ChemMate EnVision. All biopsies diagnosed for IgAN were classified according to Oxford Classification by a pathologist blinded to the clinical and molecular data.

### Immunofluorescence

Paraffin-embedded or frozen biopsy sections from patients with IgAN and healthy kidney transplants were blocked with 2% FCS, 2%BSA and goat IgG at a dilution of 1∶1000 or 1% of donkey serum. As primary antibodies we used anti-decorin (R&D Systems, Minneapolis, MN), anti-TGF-β (Abcam, Cambridge, MA) and anti-perlecan (Invitrogen, Carlsbad, CA) diluted 1∶100. Secondary antibodies were anti-goat, anti-rabbit and anti-mouse Alexa Fluor 488 (Invitrogen). Sections were mounted using ProLong Gold antifade reagent (Invitrogen) and analyzed with a Leica TCS SP5 confocal microscope (Leica Microsystems GmbH, Wetzler, Germany).Glomeruli from biopsy sections stained for TGF-β from patients with IgAN (n = 84) and controls (n = 19) were classified according to a 10 step scale in a blinded fashion. Glomeruli from frozen biopsy sections stained for perlecan from patients with IgAN (n = 45) and controls (n = 30) were analyzed using the biopix software (Biopix AB, Gothenburg, Sweden). The total area of the glomeruli was compared to the perlecan-stained area of the glomeruli. Paraffin-embedded biopsy sections were stained with PAS and hematoxylin (Merck, Darmstadt, Germany) to show sclerotic areas and histology of the glomeruli.

### Statistics

Wilcoxon's signed rank sum test was used to test differences between control and IgAN groups. Pearson's correlation coefficient was used for comparing mRNA expression to clinical data and Oxford classification data. Students t-test was used for comparing protein expression of TGF-β and perlecan in IgAN and control. Data are presented as harmonic mean ± SEM for the gene expression and mean ± SEM for TGF-β and perlecan expression in biopsies. P<0.05 was considered statistically significant.
